# Supplementary material for: Revision of the Tanzanian dicynodont Dicynodon huenei (Therapsida: Anomodontia) from the Permian Usili Formation
Source: PeerJ. 2019 Aug 22;7:e7420. doi: 10.7717/peerj.7420 (PMC6708577; doi:10.7717/peerj.7420)
Supplement: Supplemental Information 2 — List of continuous and discrete-state characters used in the phylogenetic analysis, based on that of Kammerer et al. (2019). [file peerj-07-7420-s002.docx]

**CHARACTER LIST FOR THE PHYLOGENETIC ANALYSIS**

**Continuous characters**

1. Length of preorbital region of skull relative to basal length of skull.
2. Relative length of premaxillary secondary palate.
3. Minimum width of interorbital skull roof relative to basal length of skull.
4. Relative width of temporal bar at level of postorbital bar versus the relative width at the junction of the intertemporal bar with the occipital plate.
5. Length of temporal fenestra relative to basal length of skull.
6. Relative position of pineal foramen, measured as the ratio of dorsal skull length posterior to the foramen versus dorsal skull length anterior to the foramen.
7. Height of anterior pterygoid keel in lateral view relative height of non-keel ramus.
8. Width of median pterygoid plate relative to basal skull length.
9. Angle formed by the posterior pterygoid rami.
10. Length of interpterygoid vacuity relative to basal length of skull.
11. Relative area of the internal nares.
12. Angle between ascending and zygomatic processes of the squamosal.
13. Angulation of the occiput relative to the palate, expressed the ratio of dorsal and basal lengths of the skull.
14. Ratio of length to height of mandibular fenestra in lateral view.
15. Ratio of height of dentary ramus to height of dentary symphysis.
16. Ratio of maximum height of postdentary bones (excluding reflected lamina of angular) to the height of the dentary ramus.
17. Ratio of minimum width of the scapula to maximum width of dorsal end of scapula.
18. Length of the deltopectoral crest relative to total length of the humerus.
19. Maximum width of the distal end of the radius relative to the maximum length of the radius.
20. Ratio of posterior iliac process length to acetabulum diameter.
21. Ratio of anterior iliac process Length to acetabulum diameter.
22. Length of trochanteric crest on femur relative to length of femur.
23. Breadth of scapula measured as ratio of maximal proximal width of scapula versus length of scapula (measured from dorsal edge of glenoid to proximal tip).

**Discrete-state characters**

# Premaxillae unfused (0) or fused (1).

1. Paired anterior ridges on palatal surface of premaxilla absent (0), present and converge posteriorly (1), or present and do not converge (2).
2. Lateral anterior palatal ridges absent (0) or present (1).
3. Rounded depression on anterior palatal surface of premaxilla: absent (0); present (1).
4. Posterior median ridge on palatal surface of premaxilla absent (0), present with a flattened, expanded anterior area (1), or present without a flattened, expanded anterior area (2).
5. Palatal surface of premaxilla with well-defined depressions with curved sides lateral to median ridge (if present) (0), with distinct accessory ridges lateral to medial ridge (1), or relatively flat with poorly defined or no depressions present (2).
6. Palatal surface of premaxilla with antero-posterior vascular groove lateral to median ridge (if present) absent (0); present (1).
7. Location of premaxillary teeth lateral (0), medial (1) or absent (2).
8. Posterior exposure of the premaxilla on the palate: absent (0), present (1).
9. Posterior process of the premaxilla with a non-bifurcated posterior tip (0) or with a bifurcated posterior tip (1).
10. Palatine shelf ventral to internal naris: absent (0), present (1).
11. Anterior tip of snout rounded (0), squared off (1), or with a deep central invagination, giving the snout a ‘hare-lip’ appearance in anterior view (2).
12. Marked anterior expansion of preorbital region absent (0) or present (1).
13. Snout roughly parallel to long axis of skull (0) or strongly angled ventrally (1).
14. Height of canine-bearing portion of maxilla: relatively short (0); extremely deep, with long caniniform process, but with equally long premaxilla resulting in an overall tall snout (1); extremely long caniniform process offset from rest of snout (2).
15. Snout open to back of the skull (0) or anterior margin of orbit extended posteromedially to partly close off the snout from the rest of the skull (1).
16. Septomaxilla posterodorsal spur present and widely separates nasal and maxilla (0), spur present but does not separate maxilla and nasal (i.e., nasal-maxilla suture present and well defined in this region) (1), septomaxilla spur absent (2).
17. Notch on dorsal edge of narial opening absent (0) or present (1).
18. Postnarial excavation absent (0), present, relatively small, and rounded posteriorly (1), or present, very large, and elongate (2).
19. Maxillary alveolar region short, occupying less than 53% of the ventral length of the bone (0) or tooth bearing region long, occupying 72% or more of the ventral length of the bone (1).
20. Palatal surface of premaxilla exposed in lateral view (1) or not exposed in lateral view (0).
21. Maxillary canine present as large member of tooth series (0), absent (1), or present as tusk (2).
22. Maxillary non-caniniform teeth located near lateral margin of maxilla (0), located more medially, (1), or absent (2).
23. Shelf-like area lateral to the maxillary non-caniniform teeth absent (0) or present (1).
24. Fine serrations on maxillary teeth present (0), serrations absent (1), or coarse serrations present (2).
25. Sutural contact of maxilla and prefrontal present (0) or absent (1).
26. Caniniform process absent (0) or present (1).
27. Caniniform depression: has the form of an embayment bounded by a ridge medially of palatal rim anterior to caniniform process or tusk (1), has the form of a notch in palatal rim anterior to caninfiorm process (2), or absent (0).
28. Distinct lateral caniniform buttress absent (0), present (1), or present with posteroventral furrow (2).
29. Keel-like extension of the palatal rim posterior to the caniniform process absent (0) or present (1).
30. Postcaniniform crest absent (0) or present (1).
31. Ventral edge of the caniniform process or dorsal edge of the erupted portion of the canine tusk anterior (0) to, or at the same level to slightly posterior to (1) the anterior orbital margin.
32. Nasals with a long median suture that separates the premaxilla from the frontals (0) or with a short median suture and frontals and premaxilla in close proximity (1)
33. Nasal bosses absent (0), present as a median swelling with a continuous posterior margin (1), present as paired swellings near the dorsal or posterodorsal margin of external nares (2), present as paired swellings that meet in the midline to form a swollen anterodorsal surface on the snout (3).
34. Naso-frontal suture relatively straight, interdigitated, or gently bowed (0), with a distinct anterior process (1), or with a distinct posterior process (2).
35. Transverse crest approximately at level of naso-frontal suture absent (0); present and straightly transverse to curved with posterior convexity (1); present and strongly curved with posterior concavity (2).
36. Lacrimal does not contact septomaxilla (0) or does contact septomaxilla (1).
37. Prefrontal bosses absent (0), present but separate from nasals (1), or present and confluent with nasal bosses (2).
38. Raised, sometimes rugose, circumorbital rim absent (0) or present (1).
39. Frontal contribution to the dorsal rim of the orbit: broad, frontal forms a major part of the orbital rim (0); thin or absent, if present a thin frontal process extends laterally between the prefrontal and postorbital to reach the orbital margin (1).
40. Postfrontal bone present on dorsal surface of skull (0) or absent (1).
41. Postorbital bar without (0) or with thickenings and rugosities (1).
42. Mediolateral flattening and anteroposterior expansion of postorbital bar for most or all of its length absent (0) or present (1).
43. Temporal portion of skull roof relatively straight, without a strong break in slope (0), or temporal portion of skull roof angled dorsally with a strong break in slope near its anterior end (1).
44. Preparietal bone absent (0), present and flush with skull roof (1), present and depressed (2).
45. Lateral ridges bounding preparietal absent (0) or present (1).
46. Parietals’ contribution to skull table transversely as broad as long (0), longer anteroposteriorly than broad (1), or shorter anteroposteriorly than broad (2).
47. Parietal posterolateral process slender and elongate (0), or short (1).
48. Parietals well exposed on the skull roof and relatively flat (0), parietals exposed in midline groove or channel (1), dorsal parietal exposure narrow and crest-like (2).
49. Postparietal bulges outwards as ovoid swellings at posterior end of sagittal crest: no (0); yes (1).
50. Orientation of the temporal portion of the postorbital: relatively flat, so that most of the exterior surface of the bone faces dorsally (0), close to vertical, so that most of the exterior surface of the bone faces laterally (1), or bi-planar, with approximately equally-sized dorsal and lateral surfaces that are close to perpendicular (2).
51. Postorbitals extend the entire length of intertemporal bar (0) or do not extend the entire length of intertemporal bar, such that the posterior portion of the bar is formed only by the parietals (1).
52. Fossa on the ventral surface of the intertemporal bar formed by the postorbital and parietal large (0), reduced (1), or absent (2).
53. Pineal foramen present (0); absent (1).
54. Circumpineal ornamentation: chimney-like boss (0); no boss, foramen flush with skull surface (1); dome- or collar-like boss, rugosity present (2); boss present with incomplete border, more strongly developed on lateral edges of pineal foramen (3).
55. Orientation of pineal foramen: exits perpendicular to long axis of intertemporal bar (0); angled anterior to perpendicular relative to long axis of intertemporal bar (1).
56. Interparietal does not contribute to intertemporal skull roof (0), makes a small contribution to intertemporal skull roof (1), or makes a large contribution to intertemporal skull roof (2).
57. Squamosal without (0) or with (1) a small or (2) large lateral fossa for the origin of the lateral branch of the M. adductor mandibulae externus. ORDERED
58. Distinct dorsolateral notch in squamosal below zygomatic arch in posterior view absent (0) or present (1).
59. Squamosal posteroventral process short such that there is relatively extensive exposure of quadrate and quadratojugal in posterior view and the quadrate foramen (if present) is visible in posterior view) (0) or long such that nearly all of the quadrate and quadratojugal are covered by the squamosal in posterior view and the quadrate foramen (if present) is not visible in posterior view (1).
60. Zygomatic portion of the squamosal without folded edge (0), out-turned to downturned (1) (*Oudenodon*, *Odontocylops*, etc.), or folded-over (2) (*Pelanomodon*, *Geikia*). ORDERED.
61. Dorsoventral expansion of squamosal posterior to postorbital bar: (0) absent (1) present.
62. Zygomatic process of squamosal parasagittally deep (0), narrow and rod-like (1), or transversely expanded (2).
63. Oblique ridge on lateral side of zygomatic arch giving triangular cross-section and overhanging a weak groove present (1) or absent (0).
64. Squamosal zygomatic process narrowly based and in line with occipital condyle (0) or widely based and flares posteriorly beyond occipital condyle (1).
65. Sutural contact of squamosal and maxilla absent (0) or present (1).
66. Squamosal separated by tabular bone from supraoccipital (0) or contacts supraoccipital (1).
67. Suborbital boss on jugal absent (0) or present (1).
68. Quadratojugal narrow and rod-like (0) or plate-like distally (1).
69. Quadrate with a dorsal lobe that has a convex, rounded anterior edge that rests against quadrate ramus of pterygoid (0) or with a dorsal lobe that is developed into a distinct process that extends anteriorly along the quadrate ramus of the pterygoid and is triangular to sub-triangular in shape (1).
70. Vomers unfused (0) or fused (1).
71. Mid-ventral plate of vomers with an expanded, oval-shaped area posterior to junction with premaxilla (0) or without a notable expanded area posterior to junction with premaxilla (1).
72. Mid-ventral plate of vomers relatively wide in ventral view (0), more narrow and blade-like in ventral view (1).
73. Trough on mid-ventral plate of vomers (i.e., ventral surface concave ventrally with raised edges): present (0) or absent (1).
74. Palatine dentition present (0) or absent (1).
75. Bone texture of the palatine: primarily smooth, without evidence of keratinized covering (0); relatively smooth but with fine pitting and texturing suggestive of a keratinized covering (1); rugose and textured (2).
76. Position of palatine: raised, central palatine boss present (0); entire palatine flush with surrounding palatal elements (1); raised posterior section with anterior section that is flush with the secondary palate (2).
77. Paired fossae on palatine surface absent (0); present (1).
78. Palatine widest at its approximate midpoint of length (0), widens posteriorly (1), width relatively constant for entire length (2), widens anteriorly forming a palatine pad (3). ORDERED
79. Foramen on the palatal surface of the palatine absent (0) or present (1).
80. Lateral palatal foramen absent (0), present at level of the anterior, expanded palatal exposure of the palatines (1), present posterior and dorsal to the level of the anterior, expanded palatal exposure of the palatines (2).
81. Sutural contact of palatine and premaxilla absent (0) or present (1).
82. Labial fossa surrounded by maxilla, jugal, and palatine absent (0) or present (1).
83. Ectopterygoid extends further posteriorly than palatine in palatal aspect (0), or does not extend further posteriorly than palatine in palatal aspect (1), or absent (2).
84. Ectopterygoid dentition absent (0) or present (1).
85. Pterygoids contact anteriorly (0) or separated by vomers (1).
86. Transverse flange of pterygoid projects laterally, free of posterior ramus (0), projects laterally, bound by posterior ramus (1) does not project laterally (2).
87. Anterior pterygoid keel: absent (0); present (1).
88. Anterior pterygoid keel extending for most of the length of anterior ramus of pterygoid (0); anterior pterygoid keel restricted to the anterior tip of the anteritor ramus of the pterygoid (1).
89. Contact of pterygoid and maxilla absent (0) or present (1).
90. Converging ventral ridges on posterior portion of anterior pterygoid rami absent (0) or present (1).
91. Ventral surface of the median pterygoid plate depressed (0), smooth and flat (1), with a thin median ridge (2), with a wide, boss-like median ridge (3), or with a low rugose median swelling (4), or with a conical ventral projection (5), or with thin paried ridges that are contiguous with the edges of the interpterygoid vacuity (6).
92. Pterygoid dentition present, conical (0); absent (1); present, bucco-lingually expanded.
93. Posterior edges of the interpterygoid vacuity located dorsal to the median pterygoid plate (0) or extended ventrally such that they are flush with the median pterygoid plate (1).
94. Development of the pila antotica as a rod-like process on the anterior edge of the periotic with a corresponding notch for the trigeminal never posterior to it (0), or pronounced pila antotica absent and trigeminal notch is a horizontal hollow in the anterior edge of the periotic (1).
95. Contact between periotic and parietal absent (0) or present (1).
96. Parasphenoid excluded from (0) or reaches (1) interpterygoid vacuity.
97. Basisphenoid contribution to the basisphenoid-basioccpital tubera slopes anterodorsally at a shallow angle, forming elongate ridges on the basicranium that are close to the same height as the tubera for most of their length (0), slopes anterodorsally at a steeper angle such that the parabasisphenoid conribution is still somewhat ridge-like but the portion of the ridge on the anterior surface of the tuber is more vertically-oriented (1), or is nearly vertical, forming very weak ridges if any (2).
98. Stapedial facet of basisphenoid-basioccipital tuber exposed laterally (0), exposed ventrolaterally (1), or exposed ventrolaterally and open distally (2).
99. Exposure of internal cartoid between mid-pterygoid plate and parasphenoid: directed laterally (0); directed medially (1).
100. Shape of basal tubera: bifurcating and posteriorly-directed(0); laterally directed anteroposteriorly elongate with relatively narrow edges (1); strongly rounded, such that anterior and posterior tips of tuber curve towards each other, nearly enclosing the stapedial facet; tuber inflated (2); elongate, nearly quadrangular, with tubera extremely close together (3).
101. Margin of fenestra ovalis formed predominantly by parabasisphenoid, with little or no contribution from basioccipital (0), formed by approximately equal portions of parabasisphenoid and basioccipital (1), or formed predominantly by basioccipital, with little or no contribution by parabasisphenoid (2).
102. Intertuberal ridge absent (0) or present (1).
103. Dorsal process on anterior end of epipterygoid footplate absent (0) or present (1).
104. Stapedial foramen present (0) or absent (1).
105. Dorsal process of the stapes present (0) or absent (1).
106. Tabular contacts opisthotic (0) or separated from opisthotic by squamosal (1).
107. Prootic bearing rectangular alar process that forms a plate raised above surface of temporal fenestra wall, in front of fossa: absent (0) present (1).
108. Exoccipital and basioccipital contributions to the occipital condyle distinct (0) or co-ossified into a single unit (1).
109. Occipital condyle round to subspherical in posterior view (0) or distinctly tri-radiate (1) in posterior view.
110. Circular central depression or fossa on the occipital condyle between the exoccipitals and basioccipital present (0) or absent (1).
111. Lateral edge of paroccipital process drawn into sharp posteriorly-directed process that is distinctly offset from the surface of the occipital plate: absent (0) present (1).
112. Floccular fossa present (0) or absent (1).
113. Mandibular fenestra absent (0), present (1), or present but occluded by a thin sheet of the dentary (2).
114. Jaw ramus straight in dorsal view, without strong lateral bends (0), or bends strongly laterally (1) posterior to symphysis.
115. Dentaries sutured (0) or fused (1) at symphysis.
116. Teeth present on dorsal surface of dentaries (0), medially displaced, sometimes on a swelling or shelf (1), or absent (2).
117. Fine serrations on dentary teeth present (0), serrations absent (1), or coarse serrations present (2).
118. Denticulated cingulum on dentary teeth absent (0) or present (1).
119. Anteriormost dentary tooth: not distinct from rest of tooth row (0); massively enlarged and incisiform (1).
120. Jaw symphysis terminates in dorsal platform bearing the incisors and canine elevated above level of posterior dentary ramus (0); Symphyseal region of lower jaw smoothly rounded and at same level as rest of dentary ramus in lateral view (1), with an upturned beak that is raised above the level of the dorsal surface of the jaw rami and has a scooped-out depression on its posterior surface (2), drawn into a sharp, spiky beak (3), or shovel-shaped beak with a rounded or squared-off edge and a weak depression on its posterior surface (4).
121. Curved ridge that follows the profile of the symphysis present on the edge between the anterior and lateral surfaces of the dentary absent (0) or present (1).
122. Boss present on ventral surface of anterior dentary ramus. absent (0) present (1).
123. Dentary table absent (0) or present (1).
124. Posterior dentary sulcus absent (0), present but does not extend past dentary teeth (if present) (1), present and extends past dentary teeth (if present), but is relatively wide and shallow (2), or present, extends past dentary teeth (if present) and is narrower and deeper (3).
125. Tall, dorsally-convex cutting blade on medial edge of dorsal surface of dentary absent (0) or present (1).
126. Lateral dentary shelf absent (0), present but relatively small (1), present and well developed (2).
127. Anterodorsal edge of lateral dentary shelf relatively flat (0), with a groove (1), or developed into a rounded swelling (2).
128. Lateral dentary shelf relatively thick, with distinct dorsal and ventral surfaces above the mandibular fenestra (0) or a thin ventrolaterally-directed sheet that forms the dorsal margin of the mandibular fenestra (1).
129. Splenial symphysis unfused (0) or fused (1).
130. Spenial contribution to dentary symphysis: anterior process on splenial present in ventral view (0) or absent (1).
131. Exposed contribution of the angular to the symphysis: absent (0) present (1).
132. Coronoid bone present (0), or absent (1).
133. Angular with anterolateral trough for the posterior process of the dentary absent (0) or present (1).
134. Reflected lamina: (0) reflected lamina large, rounded, unornamented; (1) with perpendicular ridges; (2) with reticulate ridges; (3) triradiate, with distinct groove-ridge-groove morphology dorsoventrally arrayed along lamina; (4) small, tab-like (more elongate than rounded), unornamented (5); large, rounded, but with only a central groove bisecting the lamina.
135. Reflected lamina of angular closely approaches or touches articular (0) or widely separated from articular (1).
136. Prearticular with (0) or without (1) lateral exposure posteriorly.
137. Articular distinct (0) or at least partially fused to prearticular (1).
138. Surangular vertical lamina present and lateral to articular (0) or absent (1).
139. Jaw joint allows strictly orthal closure (0); allows parasagittal movement with joint surfaces of quadrate and articular approximately equal (1); allows parasagittal movement with joint surfaces on articular large than that of quadrate (2). ORDERED
140. Enlarged dentary caniniform present (0) or absent (1).
141. Number of sacral vertebrae three (0), four (1), five (2), or six or more (3).
142. Number of sternal bosses: 2 (0), 4(1).
143. Cleithrum absent (0) or present (1).
144. Anterior edge of scapula extended laterally to form a strong crest (1) or not (0).
145. Origin of triceps on posterior surface of scapula relatively low (0) or developed into a prominent posterior projection (1).
146. Acromion process: absent or very small (0) or present and well defined (1).
147. Procoracoid foramen or notch entirely contained within the procoracoid (0) or formed by contributions of the procoracoid and scapula in lateral view (1).
148. Procoracoid does not participate in formation of glenoid (0) or participates in formation of glenoid (1).
149. Proximal articular surface of humerus formed by a slightly convex area on proximal surface of the bone without much expansion onto the dorsal surface (0), somewhat expanded with some encroachment onto the dorsal surface (1), or strongly developed and set off from rest of humerus by a weak neck (2). ORDERED
150. Insertion of M. subcoracoscapularis on humerus a rounded, rugose area on proximal end of humerus (0), short, pinna-like process (1); large elongate process (2). ORDERED
151. Insertion of M. latissimus dorsi at rugose tuberosity on the posteroventral surface of humerus (0) or extended into a dorsoventrally flattened pinna-like process (1).
152. Anterior and distal edges of deltopectoral crest close to perpendicular (0) or very obtuse (1)
153. Ectepicondylar foramen on humerus present (0) or absent (1).
154. Radial and ulnar condyle continuous (0) or well ossified and separate (1) on ventral surface of humerus.
155. Ulna with small olecranon process that does not extend far past the articular surface for the humerus (0), or with a large olecranon process that extends well past the articular surface for the humerus (1).
156. Distal carpal 5: present as a distinct element (0), not present as a distinct element (1).
157. Manual digit III, shape of second phalanx: short (disc-like) (0), absent (1).
158. Manual digit IV, phalangeal number: 5 (0), or 3 (1).
159. Dorsal edge of ilium: unnotched (0) or notched (1).
160. Pubic plate is significantly expanded anteroposteriorly, such that its length is comparable to that of ischium (0) or anteroposteriorly short, so that it is much shorter than ischium (1).
161. Pubic plate is significantly expanded ventrally such that it is nearly the same height as ischium (0) or reduced ventrally such that it is shorter than ischium (1).
162. Distinct cranial process on anterior end of pubis absent (0) or present (1).
163. Femoral head continuous with the dorsal margin of femur (0) or offset dorsally from dorsal margin (1).
164. Proximal articular surface of the femur present as a weak swelling that is mostly limited to the proximal surface of the bone (0) or present as a more rounded, hemispherical swelling that has some encroachment on the anterior surface of the femur (1).
165. Insertion of M. iliofemoralis present as a low rugosity on the dorsolateral portion of the femur (0), developed into a distinct crest that extends down part of the lateral surface of the femur (1) or a lateral crest that is split into a distinct first trochanter and third trochanter (2). ORDERED
166. Pedal digit III, shape of second phalanx: short (disc-like) (0), absent (1).
167. Pedal digit IV, phalangeal number: 5 (0), 4 (1), or 3 (2).
168. Pedal digit IV, shape of second and third phalanges: long (0), short (1).
169. Pedal digit V, shape of second phalanx: short (0), absent (1).
170. Greatly enlarged vascular channels present (1) or absent (0).
171. Anterior face of dentary symphysis: unornamented (0), with median ridge (1).
172. Supinator process above ectepicondyle of humerus absent (0), present (1).
173. Morphology of supinator process: low, broadly separated from the shaft (0), tall and subvertical, with dorsal margin close to base of shaft (1), discrete, tab-like process occupying restricted portion of anterior face of distal humerus (2).
